# Evaluation of computed tomography metal artifact and CyberKnife fiducial recognition for novel size fiducial markers

**DOI:** 10.1002/acm2.14142

**Published:** 2023-09-06

**Authors:** Hikaru Nemoto, Masahide Saito, Toshihiro Suzuki, Hidekazu Suzuki, Naoki Sano, Zennosuke Mochizuki, Koji Mochizuki, Koji Ueda, Takafumi Komiyama, Kan Marino, Shinichi Aoki, Mitsuhiko Oguri, Hiroshi Takahashi, Hiroshi Onishi

**Affiliations:** ^1^ Department of Advanced Biomedical Imaging University of Yamanashi Yamanashi Japan; ^2^ Department of Radiology University of Yamanashi Yamanashi Japan; ^3^ Kasugai CyberKnife Rehabilitation Hospital Yamanashi Japan; ^4^ Department of Radiology Shizuoka General Hospital Shizuoka Japan

**Keywords:** CT‐artifacts, CyberKnife system, fiducial, image recognition, markers

## Abstract

**Purpose:**

This study aimed to compare fiducial markers used in CyberKnife treatment in terms of metal artifact intensity observed in CT images and fiducial recognition in the CyberKnife system affected by patient body thickness and type of marker.

**Methods:**

Five markers, ACCULOC 0.9 mm × 3 mm, Ball type Gold Anchor (GA) 0.28 mm × 10 mm, 0.28 mm × 20 mm, and novel size GA 0.4 mm × 10 mm, 0.4 mm × 20 mm were evaluated. To evaluate metal artifacts of CT images, two types of CT images of water‐equivalent gels with each marker were acquired using Aquilion LB CT scanner, one applied SEMAR (SEMAR‐on) and the other did not apply this technique (SEMAR‐off). The evaluation metric of artifact intensity (*M_SD_*) which represents a variation of CT values were compared for each marker. Next, 5, 15, and 20 cm thickness of Tough Water (TW) was placed on the gel under the condition of overlapping the vertebral phantom in the Target Locating System, and the live image of each marker was acquired to compare fiducial recognition.

**Results:**

The mean *M_SD_* of SEMAR‐off was 78.80, 74.50, 97.25, 83.29, and 149.64 HU for ACCULOC, GA0.28 mm × 10 mm, 20 mm, and 0.40 mm × 10 mm, 20 mm, respectively. In the same manner, that of SEMAR‐on was 23.52, 20.26, 26.76, 24.89, and 33.96 HU, respectively. Fiducial recognition decreased in the order of 5, 15, and 20 cm thickness, and GA 0.4 × 20 mm showed the best recognition at thickness of 20 cm TW.

**Conclusions:**

We demonstrated the potential to reduce metal artifacts in the CT image to the same level for all the markers we evaluated by applying SEMAR. Additionally, the fiducial recognition of each marker may vary depending on the thickness of the patient's body. Particularly, we showed that GA 0.40 × 20 mm may have more optimal recognition for CyberKnife treatment in cases of high bodily thickness in comparison to the other markers.

## 1 INTRODUCTION

Stereotactic body radiotherapy (SBRT) using CyberKnife (Accuray, Sunnyvale, California, USA) is effective in treating tumors that are affected by physiological movements derived from respiration, rectal gas movement, and urine volume in the bladder and has been introduced in global institutions.^1^, ^2^ There are two irradiation techniques to treat tumors using CyberKnife: (1) irradiation synchronized with fiducial markers implanted in the body (fiducial tracking) and (2) markerless dynamic body tracking irradiation using alignment x‐ray images, that is, Xsight Lung tracking or Xsight Spine Tracking. This markerless dynamic body tracking method requires that the tumor have sufficient contrast relative to the surrounding region; for example, tumor located anywhere in the spine or near the spine and located in the peripheral lungs and apex lung region. However, the fiducial tracking method can be used for soft tissue targets, such as the prostate, pancreas, liver, and lung tumors, wherein the Xsight lung method is unsuitable.^3^ While using fiducial tracking, gold fiducial markers that are visible under x‐ray imaging are often used to align the target during the course of treatment using Target Locating System (TLS). TLS consists of an orthogonal x‐ray imaging system, and the x‐ray images involving fiducial markers are registered to digitally reconstructed radiographs (DRRs) derived from planning CT images using template matching.^4^, ^5^ Registration accuracy between x‐ray images with fiducial markers and DRRs is affected by how accurately the software can identify the location of the markers, that is, fiducial recognition. Therefore, several sizes and shapes of fiducial markers are used depending on the tumor location and the patient's body thickness. The size and shape of the marker affect fiducial recognition in the TLS and the strength of metal artifacts in computed tomography (CT) images for treatment planning. Specifically, the use of larger fiducial makers has the potential to improve fiducial recognition while also increasing metal artifacts. For treatment planning, metal artifacts make it difficult to accurately delineate the tumor and normal tissue.^6^, ^7^, ^8^, ^9^ These artifacts also have the potential to propagate to density assignment errors and dose calculation errors.^10^, ^11^, ^12^ Furthermore, implanting fiducial markers in patients with higher body thickness or in locations where they overlap with bone or other highly absorbent material may decrease their capacity for fiducial recognition. In addition, these fiducial markers are implanted using a needle, which is highly invasive and can cause pneumothorax, bleeding, and infections. Therefore, fiducial markers implanted using thinner needles are preferable. Patel et al recommended using thinner needles when implanting fiducial marker at the abdominal region.^13^ For these reasons, in order to select the optimal fiducial marker, it is necessary to evaluate and take into consideration factors such as fiducial recognition, metal artifacts, and the size of the marker's needle.

Many commercial fiducial markers are currently used. The Gold Anchor (GA) (Naslund Medical AB, Huddinge, Sweden) and ACCULOC (CIVCO Medical Solutions, Kalona, IA, USA) are commonly used in CyberKnife treatment. The needle size used to implant each fiducial marker is 25 and 18 G, respectively. Two novel GA fiducial sizes, GA0.4 mm × 10 mm and GA 0.4 mm × 20 mm, have recently been introduced for CyberKnife treatment. The novel size GA is thicker than the conventional size GA 0.28 mm × 10 mm and GA 0.28 mm × 20 mm, which may improve fiducial recognition. Additionally, the needle size of novel GA is 22 G. The novel size of GA 0.4 × 10 mm and GA 0.4 × 20 mm is thinner than ACCULOC, making them less invasive. However, there have been no reports evaluating GA 0.4 mm × 10 mm and GA 0.4 mm × 20 mm. Therefore, this study aimed to compare the strength of metal artifacts in CT images and the effects of patient body thickness on fiducial recognition in CyberKnife treatment for two conventional size (GA 0.28 mm × 10 mm, 20 mm) and two novel‐sized GAs (GA 0.4 mm × 10 mm, 20 mm) with a standard cylinder marker, ACCULOC (0.90 mm × 3.0 mm).

## 2 MATERIAL AND METHOD

### 2.1 Characteristics of fiducial markers

**FIGURE 1:**
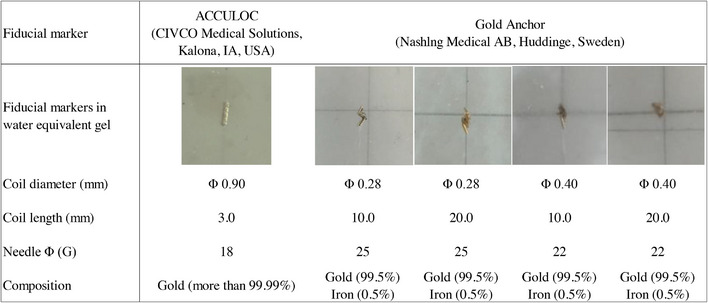
Characteristics of fiducial markers evaluated in this study.

Figure 1 presents the characteristics of the markers evaluated in this study. There are two fomethods of implanting GAs in the body: ball‐ and straight‐shaped. A ball shape is formed by inserting a coil while maintaining the position of the needle. Though the uncoiled length of GA is 10 or 20 mm, the diameter of ball‐shaped GA becomes approximately 5 or 6 mm once implanted. However, a straight shape is formed by inserting the coil while pulling the needle out. The maximum length of recognition region on TLS is 8.0 mm. Therefore, fiducial markers longer than approximately 8 mm may have been misrecognized by the CyberKnife system; as the straight‐shaped GA was 10 and 20 mm (>8.0 mm) on the TLS, only ball‐shaped GA (0.28 mm × 20 mm, 0.28 mm × 20 mm, 0.4 mm × 10 mm, and 0.4 mm × 20 mm) were evaluated in this study. These gold markers were compared with ACCULOC (0.90 mm × 3 mm), which is a cylinder‐type gold marker with pharmaceutical approval (Pharmaceutical Affairs Regulatory in Japan) for CyberKnife treatment.

### 2.2 Evaluation of CT artifacts

#### 2.2.1 Acquisition and reconstruction protocol

The CT artifacts were evaluated for each fiducial marker. First, CT images of a water‐equivalent gel (50 × 50 × 25 mm, cube‐shaped), which had each marker implanted, were acquired. All CT images were acquired using Aquilion LB CT scanner (Canon Medical Systems Corporation, Japan) with the following settings: 120 kV, 250 mA, 125 mAs, 1.0 mm slice thickness, 512 × 512 pixels and the CTDIvol was 16.6 mGy. To evaluate the effect of different reconstruction algorithms on each fiducial marker, CT images for each marker were reconstructed using the following two types of reconstruction algorithms: images with single‐energy metal artifact reduction (SEMAR; Canon Medical Systems, Japan) applied (SEMAR‐on) and images without applying SEMAR (SEMAR‐off). The SEMAR algorithm can be used to reduce metal artifacts using forward projection, backward projection, and subtracting sinogram. Previous studies described the SEMAR algorithm in detail.^14^, ^15^

#### 2.2.2 Objective evaluation of CT artifacts

The marker size in the CT image was approximately 10 pixels. Therefore, CT artifacts were analyzed in a 61 × 61 pixel axial slice centered at the center of gravity coordinates of each marker. To evaluate artifacts, the standard deviation (SD) of CT value was used as a metric for artifact intensity (*M_SD_*) defined by Equation (1) was calculated for each fiducial marker. In this study, we defined *M_SD_* as,

(1) $$M_{SD}\;\;\left[\mathrm{HU}\right]=\;\sqrt{\frac{1}{N-1}\sum_{i\;=\;1}^{N}{\left|X^{\prime }i-\;\mu \right|}^{2}}$$

where, the *X’* represents the CT value of voxels that exceed the threshold HU value, *μ* represents the average CT value of all voxels and *N* represents the number of voxels in analysis region. Therefore, *M_SD_* represents the SD of the CT values within the analysis region. The *M_SD_* was calculated on all axial slices, including fiducial markers, and the mean *M_SD_* was compared for each fiducial marker. To exclude pixels belonging to the marker itself, a threshold of 250 HU was used to eliminate these pixels from the calculation of *M_SD_*. Figure 3k‐o shows the regions of CT values >250 HU for each marker as mask images. Welch's *t*‐test was used to compare *M_SD_* between SEMAR‐on images and SEMAR‐off images and between each GA and ACCULOC. These analyses were performed using MATLAB 2021b software (Mathworks, Natick, MA, USA).

### 2.3 Evaluation of the fiducial recognition of CyberKnife system

#### 2.3.1 Objective evaluation of fiducial recognition

The fiducial recognition in the TLS for each marker was used to evaluate the accuracy of the fiducial tracking of the CyberKnife system. All CT images of each fiducial marker were acquired using Optima CT660 (GE Medical Systems, Milwaukee, WI, USA) with the following settings: 120 kV, 400 mA, 120 mAs, 1.25 mm slice thickness, and 512 × 512 pixels, and the CTDIvol of the abdomen protocol is approximately 23.25 mGy. DRR images were generated using CT images in a treatment planning system, CyberKnife MultiPlan TPS (ver. 3.2.0, Accuray, Sunnyvale, California, USA) for each fiducial marker. For marker positioning, template matching between cropped images reconstructed from DRR on TLS and live images from the system was performed. The TLS is a PC with digital image processing software, which processes the radiographic images from the flat‐panel detectors.^16^ The template image from DRR image was cropped by 10 pixels in the anterior‐posterior, superior‐inferior, and left‐right directions from the center of the selected fiducial marker, respectively. The pixel size of both template and live images is approximately 0.4 mm, and the maximum length of recognition region is 8.0 mm. The uncertainty value displayed on the TLS was used to evaluate the fiducial recognition of the fiducial tracking. The uncertainty value was calculated between the template image and live image using the similarity index, zero‐means normalized cross‐correlation (ZNCC), for a region of 21 × 21 pixels, which included a fiducial marker. Thus, the evaluation of the ZNCC region included not only the body of the fiducial marker but also the surrounding anatomy near the fiducial marker. The ZNCC was calculated using the intensity value between the template image and live image. Subsequently, the uncertainty value was defined by Equation (2).

(2) $$\mathrm{Uncertainty}\;\;\left(\%\right)=\;100-\;I_{NCC}\left(x,y\right)$$

$$\mathrm{where},\;I_{\mathrm{NCC}}\;\left(x,y\right)=\frac{\sum_{x^{\prime },y^{\prime }}^{m}\left(I_{\mathrm{DRR}}\left(x^{\prime }-x,y^{\prime }-y\right)-\bar{I_{\mathrm{DRR}}}\right)\left(I_{\mathrm{Live}}\left(x^{\prime },y^{\prime }\right)-\bar{I_{\mathrm{Live}}}\right)}{\sqrt{\sum_{x^{\prime },y^{\prime }\in R}^{m}{\left(I_{\mathrm{DRR}}\left(x^{\prime }-x,y^{\prime }-y\right)-\bar{I_{\mathrm{DRR}}}\right)}^{2}}\sqrt{\sum_{x^{\prime },y^{\prime }\int R}^{m}{\left(I_{\mathrm{Live}}\left(x^{\prime },y^{\prime }\right)-\bar{I_{\mathrm{Live}}}\right)}^{2}}}$$

The *I_DRR_* and *I_Live_* represents intensity value in a DRR image and live image, respectively. The ZNCC always falls in the range of −1 to 1. A ZNCC value of 1 indicates that the two images are completely matched. In general, an uncertainty of 40% is the clinical threshold for the CyberKnife system.

#### 2.3.2 Live image acquisition

**FIGURE 2:**
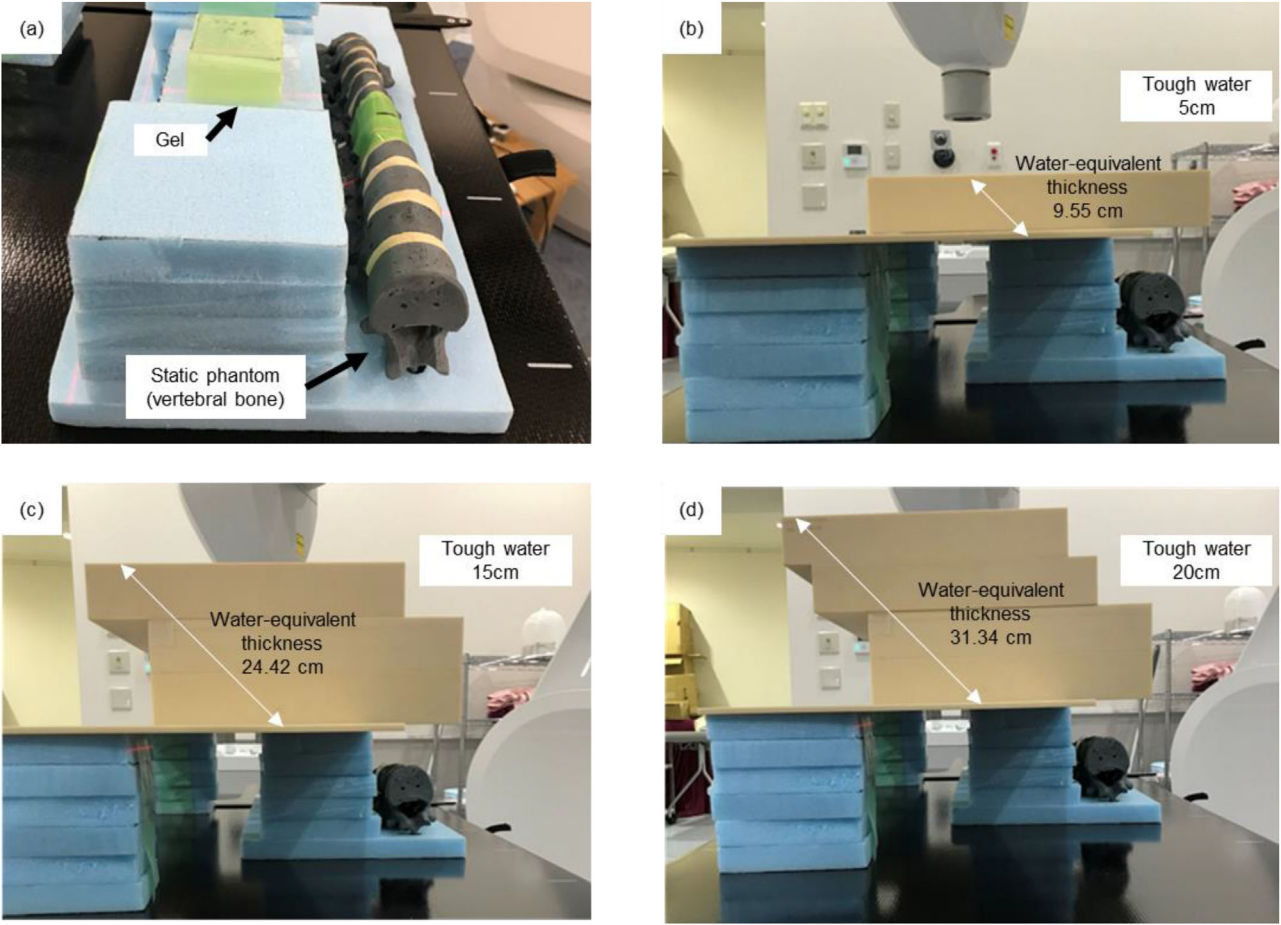
Experimental set‐up of each fiducial marker, vertebral bone phantom, and Tough Water Phantom. (a) Arrangement of water‐equivalent gel with inserted fiducial marker and static phantom (vertebral bone). The position of each fiducial marker was overlapped with the vertebral bone in TLS. (b–d) TW Phantom was placed on water equivalent gel to evaluate the effect of patient body thickness on fiducial recognition. TW thickness was following setting: (b) TW = 5 cm, (c) TW = 15 cm, and (d) TW = 20 cm.

When treating abdominal tumors using the CyberKnife (G4) system, fiducial markers often overlap with the vertebral bone. Therefore, this study evaluated fiducial recognition in the abdominal region under the condition that the vertebral phantom (Avice Inc., Tokyo, Japan) overlaps the fiducial marker in TLS. Figure 2a shows the experimental setup using a vertebral phantom and water‐equivalent gels with a marker. First, water‐equivalent gels with each fiducial marker were placed on the treatment couch where the marker overlaps with the vertebral phantom at one of the kV projection angles. Next, ten live images were acquired for each marker using the CyberKnife system, and the average uncertainty values were calculated by comparing the live and DRR images. The positioning of markers and the vertebral phantoms were made to be as identical as possible using a visible laser. In addition to the above settings, a Tough Water Phantom (TW) (KYOTO KAGAKU Corporation, Japan) consisting of 30 × 30 × 10 cm water‐equivalent rectangular materials was placed on a water‐equivalent gel to evaluate the effect of patient body thickness on fiducial recognition for each marker (Figure 2b–d). The live images were acquired under three different conditions: 5, 15, and 20 cm with the following imaging acquisition parameters: 120 kV, 100 mA, 100 ms and 130 kV, 100 mA, 100 ms; the water‐equivalent thickness of the TW on the axis from the marker to the A‐camera was approximately 9.55, 24.42, and 31.34 cm, respectively (Figure 2b–d). Statistical analysis was performed on the measured uncertainty values using IBM SPSS Statistics ver. 24 software (IBM, Armonk, NY, USA). A Wilcoxon signed‐rank test was performed on the mean uncertainty value of the ACCULOC and each GA for each condition to evaluate the fiducial recognition of each marker.

## 3 RESULTS

### 3.1 CT artifacts

**FIGURE 3:**
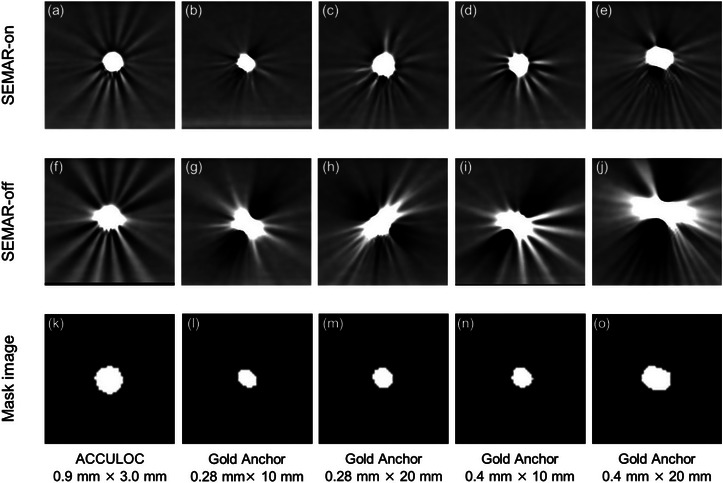
Representative axial slice of CT images for each fiducial marker. (a–e) represent CT images reconstructed with SEMAR‐on algorithm for each fiducial marker. (f–j) represent CT images reconstructed with SEMAR‐ off algorithm for each fiducial marker. (k–o) represent mask image of each marker. The masked region has intensity values greater than 250 HU in CT images.

**FIGURE 4:**
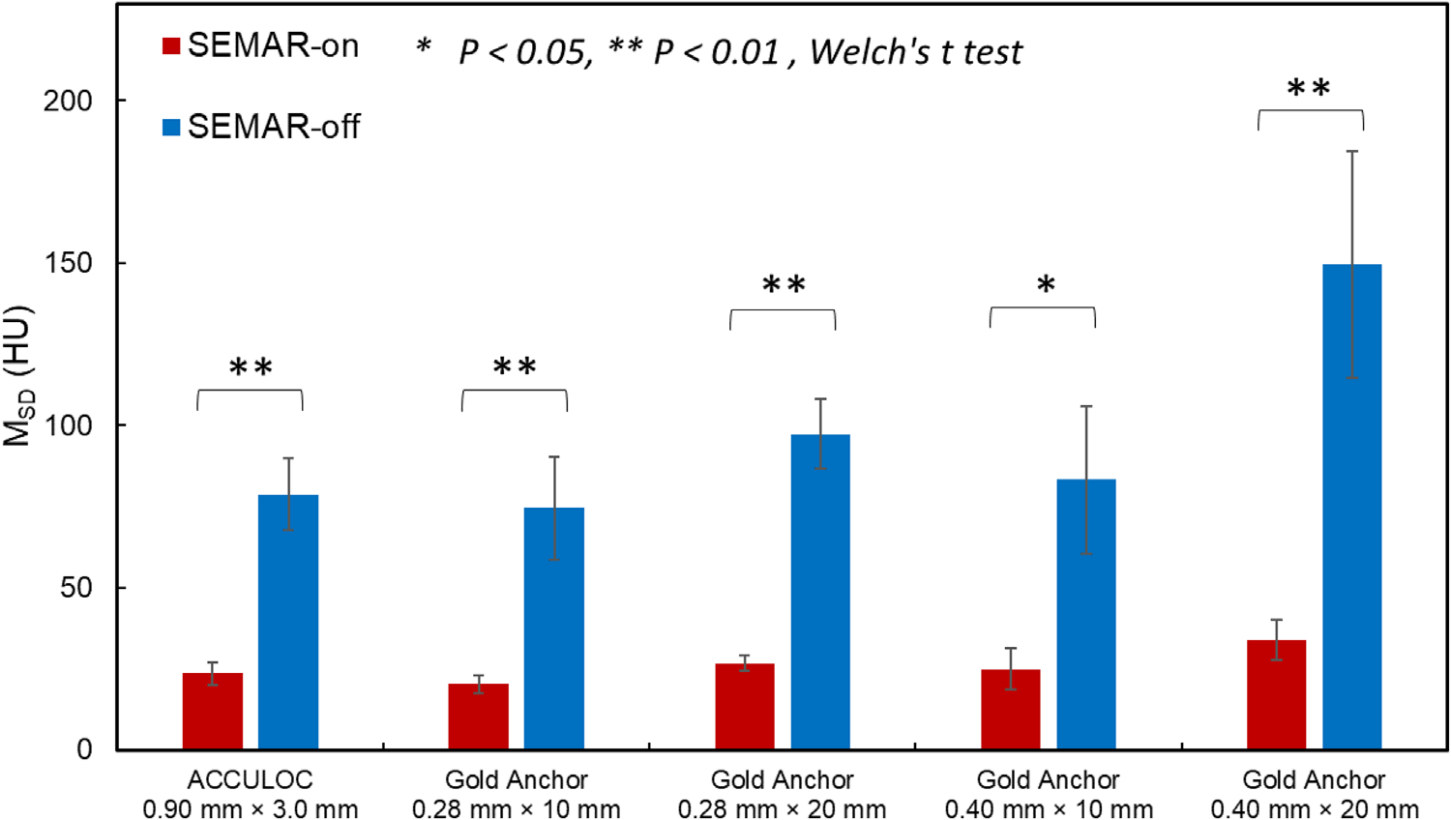
This bar chart shows the mean value of the *M_SD_* for each fiducial marker in all axial slices, including fiducial marker. The red bar represents the mean value of *M_SD_* for the SEMAR‐on CT image and the blue bar represents that of SEMAR‐off CT images.

Figure 3 shows the CT images in the axial slice for each marker, reconstructed using two types of algorithms: SEMAR‐on and SEMAR‐off. In Figure 3, high frequency streak artifacts are observed around the marker in the ACCULOC of (f). However, in the GA of (g–j), low frequency streak artifacts are extensively observed in a directional fashion, while high frequency streak artifacts are partially present in a particular direction. In severe cases like (j), metal artifacts completely obscure the adjacent water equivalent gel region. In the SEMAR‐on images, the streak artifacts were observed as shown in Figure 3a–e; however, their intensity was markedly mitigated in comparison to the SEMAR‐off images shown in (f–j). This substantially ameliorated the discernibility of the water‐equivalent gel region proximate to the marker. Figure 4 shows the mean *M_SD_* for each marker in all axial slices including the fiducial marker. The *M_SD_* of the CT values was calculated for the 61 × 61‐pixel region excluding the region of the marker (>250 HU). Regarding SEMAR‐off images, the mean *M_SD_* of all axial slices was 78.80, 74.50, 97.25, 83.29, and 149.64 HU for ACCULOC, GA 0.28 mm × 10 mm, GA 0.28 mm × 20 mm, GA 0.4 mm × 10 mm, and GA 0.4 mm × 20 mm, respectively. The *M_SD_* for GA 0.28 mm × 10 mm, GA 0.28 × 20 mm, and GA 0.4 × 10 mm did not show statistically significant differences compared to that of ACCULOC (*p* = 0.14−0.41). However, the *M_SD_* for GA 0.4 × 20 mm was significantly higher than that of ACCULOC (*p* = 0.04). The *M_SD_* for ACCULOC was lower than that of all GA except 0.28 mm × 10 mm size in the SEMAR‐off images. Concerning SEMAR‐on images, the mean *M_SD_* of all axial slices was 23.52, 20.26, 26.76, 24.89, and 33.6 HU for ACCULOC, GA 0.28 mm × 10 mm, GA 0.28 mm × 20 mm, GA 0.40 mm × 10 mm, and GA 0.4 mm × 20 mm, respectively. For SEMAR‐on images, the *M_SD_* of all GA did not show statistically significant differences compared to that of ACCULOC (*p* = 0.08−0.426). Additionally, the *M_SD_* for all SEMAR‐on images was reduced compared to that of SEMAR‐off images with statistically significant differences (*p* < 0.05).

### 3.2 Evaluation of the fiducial recognition of the CyberKnife system

**FIGURE 5:**
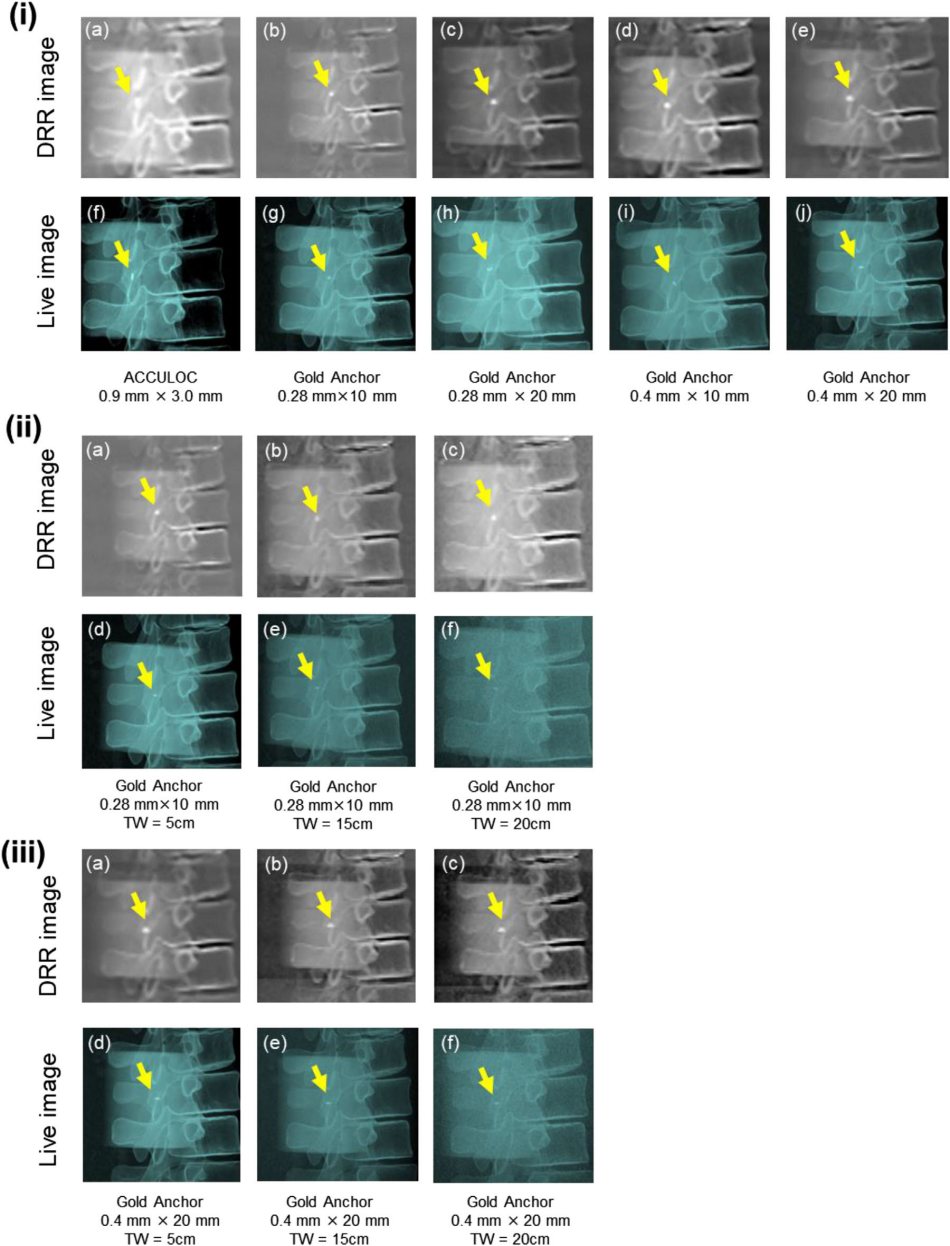
DRR images and live images with fiducial markers overlapped with the vertebral bone. The live images were obtained in the right anterior oblique angle with the following setting: 120 kV 100 mA 100 ms. Yellow arrows indicate each fiducial marker in the DRR images and Live images. (i) (a–e) DRR images of fiducial markers. (f–j) Live images of fiducial markers. Each image was acquired under the condition of 5 cm TW thickness. (ii) and (iii) DRR images and Live images of GA 0.28 mm × 10 mm and GA 0.4 mm × 20 mm under the three conditions: (a) and (d) TW thickness = 5 cm, (b) and (e) TW thickness = 15 cm, (c) and (f) TW thickness = 20 cm. GA 0.28 mm × 10 mm was the worst case in uncertainty value on TLS. In contrast, GA 0.4 mm × 20 mm was the best case in uncertainty value on TLS.

**FIGURE 6:**
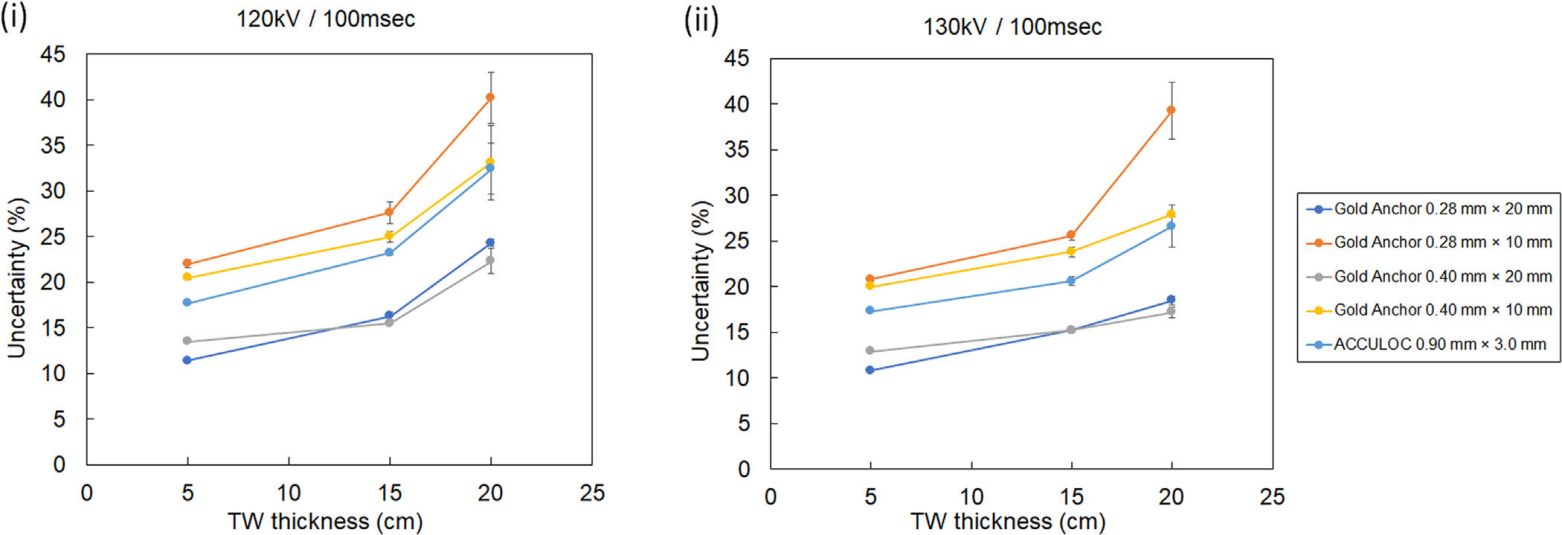
Mean uncertainty value versus Tough Water Phantom thickness for each fiducial marker. Live images of each fiducial marker were acquired with the following settings: (i) 120 kV 100 mA 100 ms and (ii) 130 kV 100 mA 100 ms.

Figure 5 shows live and DRR images for each marker acquired using the TLS of the CyberKnife system. All markers could be visually recognized in the live image and DRR images in the condition of overlapping the vertebral bone. Table 1 and Figure 6 show the mean uncertainty values and their standard deviations for 10 live images for each marker acquired under three different conditions with TW thicknesses of 5, 15, and 20 cm, respectively, overlapping the vertebral phantom. For each fiducial marker, fiducial recognition in TLS decreased with increasing TW thickness. For a TW thickness value of 5 and 15 cm, significant differences were found between ACCULOC and all GAs (*p* < 0.05). For a TW thickness value of 20 cm, significant differences were found between ACCULOC and GA 0.28 mm × 10, GA 0.28 mm × 20 mm, and GA 0.4 mm × 20 mm (*p* < 0.05), respectively, but not between ACCULOC and GA 0.4 mm × 10 mm. Fiducial recognition of GA 0.4 mm × 20 mm was significantly better under all imaging conditions and TW thicknesses compared to ACCULOC.

**TABLE 1:** The mean uncertainty value and standard deviation of each fiducial marker.

			Uncertainty (%, mean ± SD)								
			Gold Anchor								
kV/ms	mA	TW thickness (cm)	0.28 × 20 mm	*p*‐value	0.28 × 10 mm	*p*‐value	0.40 × 20 mm	*p*‐value	0.40 × 10 mm	*p*‐value	ACCULOC 0.90 × 3 mm
120/100	100	5	11.4 ± 0.1	<0.001	22.0 ± 0.4	<0.001	13.5 ± 0.0	<0.001	20.5 ± 0.2	<0.001	17.7 ± 0.1
		15	16.3 ± 0.4	<0.001	27.6 ± 1.2	<0.001	15.5 ± 0.1	<0.001	25.0 ± 0.6	<0.001	23.2 ± 0.2
		20	24.3 ± 0.4	<0.001	40.2 ± 2.8	<0.001	22.3 ± 1.4	<0.001	33.1 ± 4.1	0.508	32.4 ± 2.8
130/160	100	5	10.8 ± 0.1	<0.001	20.8 ± 0.2	<0.001	12.9 ± 0.1	<0.001	20.0 ± 0.1	<0.001	17.3 ± 0.0
		15	15.2 ± 0.2	<0.001	25.6 ± 0.5	<0.001	15.2 ± 0.1	<0.001	23.8 ± 0.5	<0.001	20.6 ± 0.5
		20	18.5 ± 0.5	<0.001	39.3 ± 3.1	<0.001	17.2 ± 0.6	<0.001	27.9 ± 2.5	0.139	26.6 ± 2.3

*Note*: Each of the fiducial marker was overlapped with the vertebral bone phantom in TLS, respectively. To evaluate the fiducial recognition of each marker, a Wilcoxon signed‐rank test was performed on the mean uncertainty value of the ACCULOC and each GA for each condition.

## 4 DISCUSSION

The fiducial recognition of markers on TLS may be significantly reduced when implanted fiducial markers overlap with bone or other high x‐ray absorbents in the body. In this study, we compared four types of GAs and ACCULOC for CT artifacts and fiducial recognition on TLS, for different patient body thickness.

Fiducial markers are commercially available in a variety of diameters and lengths. In general, the larger the size of the marker, the better the visual evaluation and fiducial recognition; however, strong metal artifacts may be generated. The generation of strong metal artifacts can significantly affect the contouring of the tumor and organs at risk^8^, ^9^ and dose calculations for treatment planning led to reported dose overestimation ranging from 9 to 39.8% in head and neck cancer treatment using linear accelerator.^17^, ^18^, ^19^, ^20^ Regarding the dose overestimation, the area in which the CT values are influenced by artifacts of the fiducial marker is limited, thus the impact on the dose distribution may be lower than the reported error. This is a concern to be addressed for future investigations. Therefore, it is important to reduce the metal artifacts caused by fiducial markers in the CyberKnife system. Our results showed that in SEMER‐off images, the *M_SD_* for GA 0.28 mm × 10 mm, GA 0.28 × 20 mm, and GA 0.4 × 10 mm did not show statistically significant differences compared to that of ACCULOC (*p* = 0.14−0.41). However, the *M_SD_* for GA 0.4 × 20 mm was significantly higher than that of ACCULOC (*p* = 0.04). In the SEMAR‐on images, all GAs and ACCULOC showed no statistically significant difference in *M_SD_*. Therefore, the results suggest that the SEMAR algorithm has the potential to minimize the intensity of artifacts caused by each marker to an equal level. Brook et al. evaluated software to improve the tumor visibility in the vicinity of fiducial markers in spectral CT, which is difficult to use in radiation therapy.^21^ Huang et al. suggest that artifact reduction allows more confident contouring of structures.^6^ Hence, in the future, fiducial markers should be evaluated under other conditions, and their impact on the accuracy of contouring the target and its surrounding structures, and dose calculations should be evaluated in more detail.

For fiducial recognition in TLS, Marsico et al. performed a subjective visual evaluation of CT images,^22^ and our previous study evaluated fiducial recognition under the conditions of difficult‐to‐track markers in the CyberKnife system.^5^ However, no study has evaluated fiducial recognition of the novel‐sized fiducial marker newly available for CyberKnife treatment. In addition, the uncertainty value is calculated for a confined 21 × 21‐pixel region, including the fiducial marker, and is affected by the x‐ray attenuation coefficient of the anatomical structures near the marker. Consequently, as the x‐ray attenuation increases, the x‐ray dose incident on the detector decreases, resulting in higher uncertainty values and decreased fiducial recognition. Specifically, increased uncertainty value was observed in patients with greater body thickness, low tube voltage in DRR imaging, and enhanced density of high‐absorbing anatomical structures, such as the pelvis and vertebral bones, which overlap fiducial markers. Therefore, this study evaluated the fiducial recognition of clinically available and novel size fiducial markers, and the impact of differences in body thickness on fiducial recognition.

For each fiducial marker, fiducial recognition in TLS decreased with increasing TW thickness. Yasue et al evaluated the fiducial marker recognition using SyncTraX FX4 (Shimadzu, Kyoto, Japan) and VISICOIL (Seti Medical, Tokyo, Japan). Their results showed a similar trend to our results. However, they reported that in the liver region, when the water equivalent thickness is greater than 25 cm, fiducial markers are more difficult to recognize, and the probability of successful tracking decreases.^23^ Our results showed that even when the water equivalent thickness was increased to 9.55, 24.42 and 31.34 cm, some good recognition was maintained for GA 0.28 × 20 mm and GA 0.4 × 20 mm particularly. Under the condition that each fiducial marker overlapped the vertebral phantom, GA 0.40 × 20 mm, and GA 0.28 × 20 mm had significantly better fiducial recognition than ACCULOC (0.90 mm × 3 mm) under all conditions (live image acquisition setting and TW phantom thickness evaluated in this study). Particularly, GA 0.40 × 20 mm had the best fiducial recognition under the condition of 20 cm TW thickness. In addition, GA 0.28 × 10 mm had an uncertainty of approximately 40% under the condition of 20 cm TW thickness, resulting in lower fiducial recognition. Therefore, we recommend a GA 0.4 mm × 20 mm to be used when the patient has higher body thickness and the marker overlaps with the vertebral bone in the TLS.

This study had several limitations. First, because the evaluation was performed using a phantom, CT artifacts, and fiducial recognition may differ depending on the difference between the shape of the phantom used and the actual patient's body shape and physiological body movements, such as breathing. Second, the shape and orientation of the implanted marker may differ depending on the person performing the implantation, which may have led to different results from those of the present study. In the future, evaluation of the effects on dose calculation under conditions, wherein the marker moves owing to the physiological displacement at other treatment sites is required. Third, we did not evaluate the effect of SEMAR on DRR image and fiducial recognition in the CyberKnife system. Fourth, using SD alone to evaluate metal artifacts in CT images may not completely evaluate the artifact severity. And we used the ROI thresholding method to eliminate regions of the marker body from the analysis region. This study used SD as a simple and quantitative measure of artifact severity that can be easily compared across different markers. Dong et al used artifact index and Zhang et al and Meyer et al used root means square error to evaluate the severity of artifacts^24^, ^25^, ^26^; thus, evaluation of metal artifact using other metric should also be considered. Finally, the ROI thresholding method may have artificially lowered the measured SD by eliminating induced streak artifacts. In this study, we used the ROI thresholding method because of the slight variation in size of the implanted ball‐shaped markers. Nevertheless, for optimal evaluation, artifacts should be assessed at consistent distance from the expected marker position.

## 5 CONCLUSION

This study evaluated the CT artifact and fiducial recognition of five fiducial markers including novel sized fiducial markers: GA 0.28 × 10, GA 0.28 × 20 mm, GA 0.40 mm × 10 mm, GA 0.40 mm × 20 mm, and ACCULOC (0.90 mm × 3 mm). We demonstrated the potential to reduce metal artifact in the CT image to the same level for all the markers we evaluated by applying SEMAR. Additionally, the fiducial recognition of each marker may vary depending on the thickness of the patient's body. Particularly, we showed that GA 0.40 × 20 mm may have more optimal recognition for CyberKnife treatment in cases of high bodily thickness in comparison to the other markers evaluated. The results of this study support the selection of fiducial markers for CyberKnife treatment.

## AUTHOR CONTRIBUTIONS

Conceptualization: H.N, M.S., and H.O. Data Curation: H.N, T.S, T.S., and K.U. Formal Analysis: H.N, M.S, T.S., and Z.M. Methodology: H.N, M.S, T.S., and H.O. Project Administration: H.N, M.S, H.T., and H.O. Resources: H.N, N.S, Z.M, K.M, T.K, K.M, S.A, M.O., and H.O. Software: H.N, M.S., and T.S. Supervision: M.S, H.T., and H.O. Writing—original draft preparation: H.N, M.S, T.S., and H.O. Writing—review & editing: H.N, M.S, T.S., and H.O. The data that support the findings of this study are openly available in Mendeley Data at https://doi.org/10.17632/c9vrmrgms3.1.

## CONFLICT OF INTEREST STATEMENT

The authors declare no conflicts of interest.
